# The Communication Chain of Genetic Risk: Analyses of Narrative Data Exploring Proband–Provider and Proband–Family Communication in Hereditary Breast and Ovarian Cancer

**DOI:** 10.3390/jpm12081249

**Published:** 2022-07-29

**Authors:** Carla Pedrazzani, Monica Aceti, Reka Schweighoffer, Andrea Kaiser-Grolimund, Nicole Bürki, Pierre O. Chappuis, Rossella Graffeo, Christian Monnerat, Olivia Pagani, Manuela Rabaglio, Maria C. Katapodi, Maria Caiata-Zufferey

**Affiliations:** 1 Department of Clinical Research, Faculty of Medicine, University of Basel, 4055 Basel, Switzerland; carla.pedrazzani@unibas.ch (C.P.); monica.aceti@unibas.ch (M.A.); reka.schweighoffer@unibas.ch (R.S.); 2 Swiss Tropical and Public Health Institute, 4123 Allschwil, Switzerland; andrea.kaisergrolimund@unibas.ch; 3 Institute of Social Anthropology, University of Basel, 4051 Basel, Switzerland; 4 Women’s Clinic, University Hospital Basel, 4031 Basel, Switzerland; n.buerki@hin.ch; 5 Oncogenetics Unit, Service of Oncology, University Hospital of Geneva, 1205 Geneva, Switzerland; pierre.chappuis@hcuge.ch; 6 Oncology Institute of Southern Switzerland, 6900 Lugano, Switzerland; rossella.graffeo@eoc.ch (R.G.); opagani@bluewin.ch (O.P.); 7 Department of Medical Oncology, Jura Hospital, 2800 Delémont, Switzerland; christian.monnerat@h-ju.ch; 8 Department of Medical Oncology, Inselspital University Hospital, 3010 Bern, Switzerland; manuela.rabaglio@insel.ch; 9 Department of Business Economics, Health and Social Care, University of Applied Science and Arts of Southern Switzerland, 6928 Manno, Switzerland; maria.caiata@supsi.ch

**Keywords:** cascade genetic screening, counselling, genetic risk, grounded theory, HBOC, intra-familial communication, proband-mediated communication, tier 1 genetic condition

## Abstract

Low uptake of genetic services among members of families with hereditary breast and ovarian cancer (HBOC) suggests limitations of proband-mediated communication of genetic risk. This study explored how genetic information proceeds from healthcare providers to probands and from probands to relatives, from the probands’ perspectives. Using a grounded-theory approach, we analyzed narrative data collected with individual interviews and focus groups from a sample of 48 women identified as carriers of HBOC-associated pathogenic variants from three linguistic regions of Switzerland. The findings describe the “communication chain”, confirming the difficulties of proband-mediated communication. Provider–proband communication is impacted by a three-level complexity in the way information about family communication is approached by providers, received by probands, and followed-up by the healthcare system. Probands’ decisions regarding disclosure of genetic risk are governed by dynamic and often contradictory logics of action, interconnected with individual and family characteristics, eventually compelling probands to engage in an arbitrating process. The findings highlight the relevance of probands’ involvement in the communication of genetic risk to relatives, suggesting the need to support them in navigating the complexity of family communication rather than replacing them in this process. Concrete actions at the clinical and health system levels are needed to improve proband-mediated communication.

## 1. Introduction

Communication of genetic risk among members of families concerned with hereditary breast and ovarian cancer (HBOC) is an essential prerequisite of cascade screening. In Switzerland, and in many countries worldwide, privacy laws mandate that the proband, i.e., the individual identified with the pathogenic variant, has the responsibility to share the genetic test results with relatives, explain implications, and advocate the use of genetic services [1,2]. However, uptake of cascade genetic screening for HBOC remains lower than 50%, suggesting that proband-mediated communication of cancer risk has significant limitations in both ensuring contact with the appropriate individuals and the transmission of accurate information [3,4,5]. Extensive literature indicates that communication of genetic risk to relatives is a difficult and complex process, compounded by a wide variety of interconnected factors that can act as facilitators or barriers. These characteristics are related to the individual (i.e., feelings about informing relatives and perceived responsibility to tell; perceived risk and disease severity; level of psychological adaptation and perceived relevance of the information; motivation to share; genetic literacy), the interpersonal dynamic (i.e., emotional closeness and frequency of interactions), the family (i.e., proximity and quality of the relationship; family forms and cohesiveness; past experience with cancer; family rules and patterns), and the community characteristics (i.e., cultural context; gender) [6,7,8,9,10,11,12,13,14,15,16,17,18]. Probands usually acknowledge their responsibility to inform relatives, but this responsibility may be experienced as a dilemma with sentiments of guilt, fear, and frustration, adding to the cancer diagnosis-associated burden [19,20]. Consequently, relatives may not be informed despite probands’ intentions to share test results, the information may be inappropriate or delayed, or, in some cases, information dissemination to relatives may be deliberately withheld [17,21,22,23].

International guidelines recommend that providers support probands in intra-familial communication of genetic risk [3,24,25,26,27]. However, this is a complex, challenging, and still limited clinical practice [3,26,27,28], likely due to the lack of information about distant relatives, lack of clarity on which side of the family harbors the pathogenic variant, and ethical and professional dilemmas about respect for patients’ autonomy and duty to warn [3,26,27,28]. Strategies used by providers to support family communication are mainly focused on information content, often delivered without continuity, or as part of research interventions rather than routine clinical practice [24].

To address the issue of poor communication of genetic test results, thus alleviating probands’ burden and increasing the accuracy of information-sharing, more active approaches are being considered, e.g., providers directly contacting relatives [3,6,24,25]. The literature suggests that provider-mediated approaches are more effective [3,6,23,25], but they have to reconcile major ethical and legal implications and comply with local legislation and with preferences about the contact modalities of all parties involved [2,3,6,24,29]. In all cases, probands’ involvement remains fundamental, e.g., consent to contact relatives, providing contact information, etc. [6,30].

In the context of the current debate regarding proband-mediated versus provider-mediated strategies to disseminate genetic cancer risk to relatives, proband-mediated communication needs to be better understood. This study aimed to clarify the process of communicating genetic risk to relatives, based on the assumption that this is an ongoing process, and that there is a “communication chain” along which information about genetic risk proceeds from healthcare providers to carriers of pathogenic variants, and from carriers to relatives. Based on this hypothesis, this study explored how healthcare providers address family communication of genetic risk with probands; how probands decide to communicate genetic risk to relatives; and how providers’ discussions with probands may affect probands’ decision to communicate genetic risk to relatives. Our study aimed to understand from the probands’ point of view, the way probands manage the process of communicating genetic risk, which begins when they receive the information about the pathogenic variant from their healthcare provider to the moment in which they decide to transmit or not transmit this information to relatives. The findings will help clarify probands’ and healthcare providers’ roles in proband-mediated communication.

## 2. Materials and Methods

This descriptive, grounded theory-based analysis used individual interviews and focus groups to collect narrative data from a sample of women identified as carriers of HBOC-associated pathogenic variants. Individuals were recruited from the CASCADE study, an open-ended cohort designed to elicit factors that enhance cascade genetic screening for HBOC and Lynch syndrome in Switzerland (NCT03124212) [31]. Confirmed carriers of pathogenic variants who were 18 years or older were recruited from university centers, cantonal hospitals, and private praxis in three linguistic regions of Switzerland. Details about the eligibility criteria and recruitment procedures have been published [32]. This study, including the collection of narrative data, was approved by appropriate ethics committees (BASEC 2016-02052).

Data collection took place between April 2019 and November 2021 among 104 probands willing to provide narrative data. The research team decided on a pragmatic data collection process, oriented by the emerging results (theoretical sampling) and in order to diversify the sample in terms of clinical history (cancer-affected or cancer-free), linguistic area (Swiss German, French, Italian), and age groups (≤39, 40–49, 50–59, ≥60). Data collection included both focus groups and interviews that were conducted either face-to-face or online. Initially, the research team planned to conduct a series of focus groups to facilitate the expression of experiences related to family communication, taking advantage of potentially synergistic effects of participants’ interactions [33]. However, some participants expressed their preference towards individual interviews for privacy reasons. Moreover, the organization of focus groups with compatible individuals was difficult due to the geographical distance among centers from similar linguistic regions, and eventually the COVID-19 pandemic. The research team developed strategies to ensure data quality and comparability, paying attention to the advantages of each data collection technique, and facing the challenges of online data collection on sensitive topics [34].

Interviews and focus groups were conducted by four members of the research team experienced in qualitative research and fluent in the language of conduction. The data collection guide was developed in English to support a common approach and then was translated in German, French, and Italian. The data collection guide asked individuals to reflect on how healthcare providers addressed genetic risk communication to relatives; how they acted upon and experienced family communication; how they perceived that communication with healthcare providers affected their own communication with relatives. According to grounded theory, data collection and analyses were conducted simultaneously until data saturation (iterativity) [35]. The research team adapted the interview guide according to the data collection mode (focus groups versus interviews, face-to-face versus online) and modified it according to emerging themes. Interviews and focus groups were audio- or video-recorded and transcribed verbatim. Pseudonyms were used for confidentiality.

Data analyses were conducted by the four interviewers and two senior researchers using the method of constant comparisons [35]. Focus groups were analyzed first to identify the topics that emerged in the discussion, and then individual interviews were addressed to gain a deeper understanding of the experiences. Each interviewer was responsible for making a first analysis of their own data, starting by reading the transcripts multiple times to become familiar with the content and to identify meaningful quotes. Data from both interviews and focus groups were continuously compared to form categories and to find relationships among concepts. Each interviewer inductively coded the data, linked the codes and grouped them into larger categories and concepts, and organized them into different topics. The prominent themes of each interview or focus group discussions were stressed and contrasted with those of other interviews or discussions, considering the characteristics of the data collected with the different techniques (in-depth vs. extensive) [33]. Regular meetings regarding emerging patterns ensured analytical validity. Disagreements in interpretation were addressed through discussion and by making constant references to the transcripts. A transversal analysis helped develop a more general understanding of the studied phenomenon. Once data saturation was achieved [35], the research team consensually developed a detailed codebook in English. Each researcher was in charge of re-coding their own data following the codebook, identifying meaningful quotes, and translating them in English. Members of the research team provided feedback and checked the relevance and the validity of the developed argumentation and the selected quotations.

## 3. Results

The findings are based on narrative data collected from 48 individuals, of whom 20 participated in individual interviews and 28 in 11 focus groups. Data collection for 7 interviews and 2 focus groups took place face-to-face, at individuals’ home or in a university classroom, while the remaining 13 interviews and 9 focus groups took place online due to COVID-19 restrictions. Table 1 presents the sample characteristics.

Individuals were on average 7 years post genetic testing. The majority self-identified as White European, married or partnered, and had at least some college education. Approximately two out of three individuals had one or more previous cancer diagnoses.

### 3.1. Communication between Healthcare Providers and Probands: Situational Challenges

The first link in the “communication chain”, which supposedly brings genetic information from providers to relatives, takes place during probands’ post-testing consultation. At that moment, probands become officially aware that they carry a pathogenic variant, that the genetic predisposition involves their relatives, and that there is an ethical and medical need to inform them. Individuals highlighted the challenging nature of these discussions. Data show variability and complexity, particularly in the way the need for family communication was broached by providers, was received by probands, and was followed-up by the healthcare system. Table 2 provides supporting quotes about communication between providers and probands.

#### 3.1.1. Variability in the Approach to Family Communication

Experiences about how the need for family communication was addressed by providers were diverse and multifaceted. About half of the sample perceived that the topic of family communication was approached in a rather superficial, hasty, and abstract manner. Consequently, the discussion did not have any concrete effect, and left them feeling “alone” in their duty to inform relatives. Details, such as information about whom to inform and whom not, were frequently missing, and if they were addressed, they were not always clear (quotes 1 and 2). One participant mentioned that she would have preferred more incisive instructions, leaving no room for uncertainty, and a more active and direct role of providers in supporting her in the process (quote 3). Other experiences were positive, describing an exhaustive discussion on family communication and a helpful attitude from providers (quote 4). Concrete interventions, such as a letter to distribute to relatives, were also considered helpful to make family communication easier and more effective (quote 5). This variability of experiences highlights that there was no common approach in addressing family communication, and that these discussions were guided primarily by providers’ individual sensitivity and interpretation of the situation.

#### 3.1.2. Difficulty in Receiving Information about Family Communication

Often individuals did not remember whether providers mentioned family communication, or they had a vague memory of it. This may be due to the amount of information given during the post-testing consultation and the particularities of this moment. Some individuals recognized that their own psychological state influenced their levels of attention and understanding (quote 6). Often counselling took place when priorities were not focused on the need to communicate genetic risk to others, particularly for cancer-affected women, who tended to focus the discussion on disease management in light of its genetic nature. Providers and probands were thus swayed away from the issue of family communication (quote 7). Even for women not being diagnosed with cancer, worries about the pathogenic variant and about decisions for risk management took precedence over the urgency to address family communication (quote 8). Therefore, both the particularity of the situation and individuals’ choices to give priority to other aspects during genetic counselling seem frequently to place family communication on “the back burner”.

#### 3.1.3. Inconsistency in the Follow-Up of the Issue of Family Communication

The possibility to discuss again the topic of family communication with healthcare providers is crucial (quote 9). Given the amount of information provided during genetic counselling, the possibility of asking questions about issues that were not clear or were poorly remembered helps probands clarify their own doubts and provide more accurate information to relatives (quote 10). Individuals highlighted how, over time, new needs and new questions arise due to changes in their own situation. However, they did not have another opportunity to discuss the topic of family communication with their providers (quote 11). In most cases, neither the genetic specialist nor other providers addressed the topic of family communication after the post-testing consultation. In the rare events that this happened, there was no coordination among providers from different specialties (quote 12). Theoretically, individuals have the possibility to contact the genetic specialist at a later point; however, most women had difficulties in taking this initiative. Some looked for information elsewhere, risking experiencing even more inconsistency in the way family communication was addressed (quote 13).

### 3.2. Probands’ Decision-Making Regarding Family Communication: Multiple Logics of Action

The second link in the communication chain involves probands’ decision to disclose genetic testing results to relatives. This decision does not take place in a single moment, and it is not linear. Different logics come into play to guide the decision to communicate or not, and the way and time to do it. These logics of action help in understanding communication or lack thereof, and are linked to the proband’s rationality, values, and beliefs. They may be more or less explicit, contradictory, and they may come into play simultaneously, making the decision-making process complex and difficult to interpret. Our data show four main logics of action as the basis of this process. Table 3 provides supporting quotes about probands’ decision-making for family communication.

#### 3.2.1. Responsibility

Discovering a pathogenic variant allocates the burden of communication with relatives to the individual immediately. There is a sort of “normative” pressure that encourages the individual to communicate genetic risk to relatives. Most individuals felt responsible to inform relatives and make them aware about their possible genetic risk. Communication of genetic information appears as a “due act”, something necessary to do, despite the implied difficulties (quotes 14 and 15). This sense of responsibility seems particularly prominent with close relatives and when there is emotional proximity. This leads to prompt and more insistent information, even if this is often more difficult due to the level of personal involvement (quotes 16 and 17). For some individuals, this sense of responsibility is also directed towards distant relatives, and even towards increasing dissemination of genetic information to the lay public. For these individuals, transmission of genetic information is crucial due to their highly developed sense of civic duty (quotes 18 and 19). Family and personal experiences may reinforce this sense of responsibility, especially when there is personal or family illness, which emphasize the importance of making others aware of the genetic risk as soon as possible (quote 20). Providers may also reinforce the sense of duty to inform relatives, encouraging or even convincing individuals to take concrete actions (quote 21).

#### 3.2.2. Self-Preservation

Communicating genetic risk to relatives inevitably implies a process of self-disclosure. Individuals not only communicate that the pathogenic variant is running in the family but also convey information that they do not necessarily want to transmit, including their own clinical condition, their fears, wishes, emotional experiences, etc. It is extremely difficult, if not impossible, to control the flow of information when communicating genetic risk. Conceivably, one may try to avoid possible discomfort and disadvantages arising from this process. Some individuals did not feel comfortable talking about genetic risk, or even perceived this process as harmful for themselves. This was especially true for cancer-affected women, where being sick was considered a private issue and sharing with relatives was not anodyne (quotes 22 and 23). According to the logic of self-preservation, probands may decide to inform relatives about the possible genetic risk but only after they feel able and comfortable to do so, for example, after completing cancer treatment and being more confident about their illness trajectory (quote 24). This sense of self-preservation emerged particularly with distant relatives when there is a geographical and emotional distance. Not knowing others’ reactions or anticipating stressful and difficult to manage responses reinforced this logic (quote 25).

#### 3.2.3. Protection of Others

Receiving information about the possibility of carrying a cancer-causing genetic variant is not a neutral event. Some probands may decide to protect relatives from the negative effects of finding out about their potential cancer risk. This decision is based on the conviction that genetic information may create more problems than advantages to the relative, at least in particular moments. This is frequently due to the anticipation of relatives’ emotional responses, and based on the interpretation of different factors, such as their personality traits, age, stage of life, or family dynamics. One may decide to postpone the transmission of genetic information because they consider their relative as particularly anxious or vulnerable, or because the relative goes through a difficult period in their life (quotes 26 and 27). The interpretation of what might hurt relatives and how to protect them provides crucial input into the logic of protecting others (quote 28), which seems particularly present with close relatives, when reactions to genetic information can be better anticipated, and the desire not to cause harm is especially prominent (quote 29).

#### 3.2.4. Respect of Autonomy

Most individuals stated that they wanted to respect relatives’ right of privacy and intimacy, meaning that the eventual diversity of opinions about genetic risk are recognized and respected. Individuals accepted that there may be good reasons for choosing not to be open to receiving genetic information, based on the conviction that everybody is a unique individual and as such must be acknowledged (quotes 30 and 31). Respect of relatives’ autonomy may lead to a lack of communication about genetic risk when the relative showed no interest or showed interest in a very superficial way and without revisiting the topic. This was especially true under the assumption that the relative has a high level of health literacy and thus does not need further information (quote 32). The logic of respecting others’ autonomy is supported by the value attributed to the free will of the other, but it is attenuated by the sense of efficacy of genetic testing or by a personal or family history of illness. Awareness of the burdensome nature of cancer and of the opportunity for prevention afforded by genetic testing tended to make it difficult to accept relatives’ choice not to engage in discussions about genetic risk (quote 33).

### 3.3. Proband-Mediated Communication: The Complexity of Arbitration and the Urgency of Support

The logics of action that guide probands’ decision to communicate or not genetic risk to relatives are multiple and dynamic, depending on the relative, the situation, the time, anticipated reactions, etc. We observed interconnections between several individual, interpersonal, family, and community characteristics commonly known to influence family communication, such as the gender, age, and life stage, and the socioeconomic and sociocultural context of relatives; probands’ personal and family experiences with illness and genetic testing; personality traits of both the proband and the relative, their levels of health literacy, and their geographical and affective distance; and family dynamics. Table 4 presents these individual and family characteristics. Table 5 provides supporting quotes about the complexity of the arbitrating process.

The same characteristics may support different and contradictory logic, and this may disorient or even paralyze the proband. For instance, in emotional proximity, the sense of responsibility frequently conflicts with the logic of protection of others or respect of their autonomy. Some individuals wanted their close relatives not to lose the opportunity to prevent cancer, but at the same time they did not want to scare them or force their choices. This ambiguity may be a difficult burden to afford (quotes 34 and 35). The sense of responsibility frequently conflicted with the logic of self-preservation for cancer-affected probands, who may feel the duty to inform relatives but at the same time also feel the potentially inherent burden of talking about themselves. It is also possible that logic may support one another, strongly encouraging the proband to adopt specific communication behaviors (quote 36). For example, the underestimation of cancer risk for male relatives by both the proband and the relative may attenuate the sense of responsibility and reinforce respect for the autonomy of others (quote 37). Logic comes into play simultaneously and interacts continuously, leading to a high level of complexity and a real “dilemma” that forces the proband to engage in a process of arbitration. Life situations keep changing and probands have to constantly navigate variable and complex dynamics, along with frequent difficulties and feelings of inadequacy (quotes 38 and 39). The difficulty of managing communication with each relative is compounded by the need to harmonize communication at the family level. Communication with a close relative cannot but influence the decision to communicate with other relatives due to their pre-existing relationships, adding significant complexity to the management of communication.

Finally, our data show that the logic of responsibility was reinforced with forthright communication from healthcare providers. We did not identify another direct interconnection between providers’ discussion on family communication and the logics adopted by probands, although supportive and continuous communication or concrete interventions from providers could help probands deal with the difficulties arising from the arbitrating process (quotes 40 and 41).

## 4. Discussion

This study focused on proband-mediated communication of genetic risk by exploring the “communication chain” from probands’ perspective, namely how genetic information proceeds from healthcare providers to probands and from probands to relatives. We analyzed two main links of this chain: the communication between healthcare providers and probands, and probands’ decisions about disseminating genetic information to relatives.

In the first link of the chain, consistent with other studies [3,21,26,27,28,36], our results show that supporting proband-mediated communication of genetic risk is a complex process, challenges providers, and has limited applications in clinical practice. Namely, we identified three levels of complexity related to (1) the way family communication is addressed by providers, namely lack of standardization implying potential inequalities in care delivery despite extensive international recommendations [27]; (2) the decisive role of the clinical situation and probands’ priorities in receiving information about family communication, which was not addressed in previous studies; and (3) the way the process is followed up and supported by the healthcare system. We identified a fragmentation of services and lack of continuity and coordination also confirmed by others [24,26].

Concerning the second link of the chain, our study introduced a specific and different perspective of analysis, focusing on the rationale behind the probands’ decision-making process. Many individual, interpersonal, family, and community characteristics acting as facilitators or barriers to family communication were identified similarly to other studies [16,17,18,37], but they are differently interpreted through the prism of the dynamic and often contradictory logic of action behind the communication decision. What emerges is a high level of complexity in the arbitrating process of family communication, leading to poor communication of genetic risk within the family, which has also been confirmed by others [17,21,22]. Probands are usually alone in this process due to the critical aspects identified in the first link of the communication chain, risking experiencing disorientation or paralysis in the actions to be taken.

The criticalities seen in the first and second links of the “communication chain” apparently suggest an opportunity for introducing provider-mediated forms of communication. More active and direct approaches adopted by healthcare providers could potentially simplify and facilitate this process, enhance standardization, and promote equity by enabling relatives’ access to reliable and accurate information [3,6,23,25]. However, our own data, and data from an independent Swiss-based sample [38], suggest that although probands embrace some forms of provider-mediated communication, they do not want to be excluded from this process. Survey data collected as part of the CASCADE study indicate that only one in three or fewer individuals with HBOC- or LS-associated pathogenic variants endorsed provider-mediated communication [31]. Other studies have also shown a preference for proband-mediated family communication [11,22,39]. This is understandable, since communication of genetic risk may have crucial consequences for one’s life and probands are the only ones that can modulate the logic of communication in a sustainable way.

These considerations suggest that communication of genetic risk should not be oriented towards replacing probands in this process but rather in supporting them when navigating the different logics and the complexities of the situation, empowering them to reflexively construct their own decision from a range of available options. The concept of “relational autonomy” may be of particular interest to highlight the nature of the support needed in this particular context [40]. Emphasis should be placed not so much on independent choice but on choices that promote probands’ autonomy, where autonomy means self-governance and interdependence [40,41]. It is necessary to help probands understand what is important for themselves and their social network, clarify the implications of the different choices, set priorities, and consider strategies to manage the competing logics. In the era of personalized healthcare, where patient-centeredness is increasingly becoming more valuable, our data suggest the necessity to support probands with tailored interventions rather than replacing them in their role as communicators.

Our study has some limitations. First, the analysis of the “communication chain” was based only on the perspective of probands and, for the sake of sample homogeneity, individuals were only women with HBOC-associated pathogenic variants. Future studies should also address the perspectives of providers and relatives. Examining the perspectives of male probands will uncover gender-based similarities and differences. Individuals were, on average, 7 years post genetic testing, which may have affected their memories of their own experiences but also allowed a more neutral and accomplished view. In contrast to other studies [37], the topic of the cost of genetic testing did not emerge from our data as a barrier to risk disclosure. This is probably because 100% of the cost of genetic testing is covered by the basic health insurance in Switzerland. Finally, our findings may not extend to other hereditary conditions. Despite these limitations, this study provides an in-depth insight into the process of intra-familial communication of genetic risk. This is due to the specific hypothesis that guided our analysis, namely the existence of a “communication chain” with the proband at its center. The large sample from three linguistic regions of Switzerland and the involvement of several researchers in data collection and analyses enriched the interpretation process. These results may be extended to other countries with similar legal conditions (privacy laws) and social context (national healthcare system, family solidarity). On the contrary, cultural differences, which we could not explore in our study due to the homogeneity of the sample, may influence the logics of communication and their interactions.

In conclusion, our study suggests the need to adopt a patient-centered approach focused on the continuum of care and fostering therapeutic relationships and dialogue that empower probands to clarify relevant issues, set well-considered priorities, develop strategies to reduce contradictions, and ultimately manage family communication. Considering the concept of “relational autonomy”, a patient-centered approach must consider the realm of the family and a systematic approach that includes this complex social system [42,43]. As suggested from our results, relatives’ involvement in a family-centered approach needs an active and leading involvement of the proband who can thus act “interdependently” in the respect of their autonomy [42].

Our study’s findings suggest improving family communication through concrete actions. At the healthcare system level, provider education and standardization of procedures through guidelines are critical for improving continuity, personalization, quality, and equity in communication from healthcare providers. Providers, better addressing family communication dynamics, can help probands understand and govern communication logics and frame genetic information as useful news rather than bad news. Additionally, a “communication manager” could assist probands in navigating through these lifelong challenges and the system over time, promote genetic literacy among the lay public, and foster family communication and the implementation of cascade genetic screening. Finally, the development of instruments for tailored communication (children, men, distant and close family, etc.) and technology-mediated communication has to be accelerated to fill in existing gaps, also facilitating family-centered communication approaches [44].

## Figures and Tables

**Table 1:** Socio-demographic and clinical characteristics of the sample.

Characteristics	N = 48 (%)
**Age**—mean (SD)	51.8 (10.9)
≤39	6 (12.5)
40–49	16 (33.3)
50–59	12 (25)
≥60	14 (29.2)
**Ethnic group**	
WhiteEuropean	38 (79.2)
Ashkenazi Jewish	4 (8.3)
Asian Unknown	1 (2.1) 5 (10.4)
**Marital status**	
Married/Partnered	38 (79.2)
Divorced/Separated Single	6 (12.5) 3 (6.25)
Widowed	1 (2.1)
**Education**	
≤High school/Technical school	14 (29.2)
Some college/Complete college	14 (29.2)
University/Post-graduate degree Unknown	19 (39.6) 1 (2.1)
**One or more previous cancer diagnosis**	29 (60.4)
**Linguistic region**	
French	27 (56.3)
German	14 (29.2)
Italian	7 (14.6)

**Table 2:** Supporting quotes about “Communication between healthcare providers and probands”.

Themes	Quotes	Supporting Quotes
Variability in the approach to family communication	1	*“In the department they told me: “You have to communicate with your family …”. But it was a bit abstract. I mean, I would have left from there and I might have done nothing too …”* (Anna, 48 y.o., cancer diagnosis)—FG *
	2	*“Actually, the communication to the family was delegated to me. (…) Perhaps it was implied, they spoke more in the feminine, then for the offspring, they spoke in the masculine. (…) This thought made me think that there was no need to tell to my uncles. I understood so … but then it is the perception.”* (Carla, 48 y.o., cancer diagnosis)—I **
	3	*“No, that communication on her side (the genetic counsellor) was just too soft. And that applies to the family clarification as well, exactly the same. It shouldn’t be “it would be best to inform your relatives”, but: “We request you to clarify your family status.” Clearly and unambiguously described. Not “you could”. But: “Go there! Do it!”* (Penny, 52 y.o., no cancer diagnosis)—I
	4	*“(The physician) was absolutely available afterwards. I didn’t feel the need to see him again. Anyway, he’s a great person, I really found him to be totally adequate.”* (Katarina, 33 y.o., no cancer diagnosis)—FG
	5	*“I received a letter from the hospital explaining what it was and that I could possibly have the gene mutation and that I should contact Dr… And that’s what we did, together with the sister. Afterwards we had all the genetic meetings with her. She (the physician) explained it very well. So, for me it was never the case that I was somehow all alone and badly informed.”* (Rose, 50 y.o, no cancer diagnosis)—FG
Difficulty in receiving information about family communication	6	*“The oncologist, I can’t tell you right now if she’s been talking to me about the mutation running in the family, I don’t know. (…) When I was with her for the first time, I wasn’t doing so well psychologically.”* (Antonia, 33 y.o., no cancer diagnosis)—I
	7	*“Because of the speed with which everything happened, it (the topic of family communication) was touched on but not explored. It was said that there was a possibility to communicate to the boys and close family members, as there was heredity. This was communicated. (…) It was probably enough at that moment. Because you’re in a situation of turmoil (…) Maybe it would have been different, if illness happened afterwards.”* (Carla, 48 y.o., cancer diagnosis)—I
	8	*“So, for me the shock of finding out that I had this mutation was even greater than finding out to have a cancer. I did the test, and I got the results. It was terrible for me because it meant that I could have passed on this mutation to my daughter, and I felt guilt.”* (Luise, 45 y.o., no cancer diagnosis)—FG
Inconsistency in the follow-up of the issue of family communication	9	*“I’m really starting to get into it (communication to children) now. Before I was more about saving my own skin, that’s done, for now anyway, and now I want to save my kids.”* (Mari, 42 y.o., cancer diagnosis)—I
	10	*“No, let’s say they gave me a lot of information all at once at the beginning, so understanding and remembering everything was a bit of a struggle. (…) So, I remembered this thing, I told them (family members), but I didn’t remember it specifically. Today I came, I spoke again about this thing here (with the physician) because I had not well understood it (…) I could resume some aspects that I had not understood, because it is not obvious on so many things to understand them all obviously.”* (Sabina, 52 y.o, no cancer diagnosis)—I
	11	*“He (the physician) did talk to me about all of this, but it was rather at the beginning. So sometimes I think it would have been necessary to take up the subject again later on. Because I was just informed by him once I had gotten the result, and I didn’t really have any questions until later.”* (Gisela, 46 y.o., no cancer diagnosis)—FG
	12	*“It was mainly the geneticist who encouraged me to talk to the family. Then when I went back to my gynecologist, he asked me if I had other family members, how they had taken it. Just out of interest. But...more than out of medical concern.”* (Christine, 47 y.o., no cancer diagnosis)—FG
	13	*“I might have been able to go to him again, but somehow I looked for (information) then in other places.”* (Gisela, 46 y.o., no cancer diagnosis)—FG

* (FG) Focus Group; ** (I) Interview.

**Table 3:** Supporting quotes about “Probands’ decision-making regarding family communication”.

Themes	Quotes	Supporting Quotes
Responsibility	14	*“Communication is a due act, in the sense that (…) it is right and proper to talk about it. (…) I feel like I did the right thing. That I communicated. (…) in my opinion this (communication to relatives) is a right thing.”* (Sabina, 52 y.o, no cancer diagnosis)—I *
	15	*“I did my part. I explained to them (my relatives) what had happened to me. What could possibly happen to them... Or not. I hope it never happens to them. But I thought it was important to communicate on the subject. (…) It has been a burden on me that. I mean it’s not easy, to take the step, to do that, it’s hyper personal anyway…”* (Anna, 48 y.o, cancer diagnosis)—FG **
	16	*“Genetic risk is part of my life and our life. For me what was very important was that my family knew about it. I have a sister who tested positive (…) she’s much younger than me, she’s 13 years younger, so she was tested a few years ago. So, for me it’s very important that she knew that there was this risk.”* (Perla, 50 y.o., no cancer diagnosis)—FG
	17	*“To the people you care about, you want to say it despite this difficulty... with a person that you know and that you care about, it is more difficult to do because emotionally you are more taken... (I felt bad) for my sisters because they have children, they have nieces and nephews, so the more people you care about, in my opinion, the more difficult it is to say it.”* (Sabina, 52 y.o, no cancer diagnosis)—I
	18	*“The responsibility in the family is so needed. That’s not modern, nowadays people are no longer responsible for the cousins, grandparents, the widowed aunts, it’s not like it used to be. This is something (genetic risk) that I have to actively tell people, and I think it’s also something that should be emphasized by the authorities. This is a problem in our society.”* (Penny, 52 y.o., no cancer diagnosis)—I
	19	*“I almost felt a little responsible for bringing this to the public. (…) Simply when I got into a conversation with someone, I actually communicated it openly because I think the more we know about it, the better. And yes, the way we were actually badly informed, that doesn’t help anyone or anything.”* (Gisela, 46 y.o, no cancer diagnosis)—FG
	20	*“This is what I said to myself, I have this thing that is not good, how can I make it useful? Communicating it as my mother did with me, it came to my mind afterwards, as an information to have. Then everyone has their own time, and maybe like me you do it in stages. But it’s important to give the information so that everyone can decide what to do next. In a certain sense it’s not pleasant, it’s not easy, it’s not nice, but it’s useful information to know in order to make informed choices and not to say “if we had known about it before …”* (Sonia, 34 y.o., no cancer diagnosis)—I
	21	*“I saw the psychologist to help me deal with the situation. And then she told me about it (communication), saying: “Now you have to communicate, you have to talk about it”. … And so, it was she who … convinced me to do it.”* (Anna, 48 y.o, cancer diagnosis)—FG
Self-preservation	22	*It’s not that I go to take all the relatives and “You know I had this”. ”I hang out with a lot of people but nobody knows about my illness.”* (Bruna. 67 y.o., cancer diagnosis)—I
	23	*“It was difficult to communicate that I was ill. (…) So only my sister knew and I only decided to tell my parents when I got home. Also, because I spent 3–4 days crying all day long (…) It was clear that I was ill but I didn’t... I didn’t say it because I was mad as hell, honestly, I was mad at the world. I didn’t want to say it out loud so it became reality even if it was reality. (…) The looks of pity as if I were going to die at any moment. I won’t say... maybe because of those looks I never said it.”* (Fiona, 32 y.o., cancer diagnosis)—I
	24	*“After my chemo (I wrote to my relatives). It was not possible before, I was so weak that it was not possible. But I did it maybe a year and a half after the cancer was discovered … When I started to get better …”* (Anna, 48 y.o., cancer diagnosis)—FG
	25	*“So, it’s difficult to talk to someone who you do not have any kind of contact with—because I know I had some distant relatives in Italy somewhere. And we didn’t want to call them, since they are too far away. We tried to tell someone in the extended family who was closer to them, so that they could then transmit it. But really, with people who I barely know, I just do not feel comfortable to call them and confront them with something like that.”* (Gisela, 46 y.o., no cancer diagnosis)—FG
Protection of others	26	*“I never talked to my sister, I don’t even know how she reacted (to my situation). She is scared (about cancer). She’s really scared. She’s always been afraid.”* (Clara, 48 y.o., cancer diagnosis)—I
	27	*“I decided to inform only my cousins and not my uncles or aunts because of their age. I felt it would be “too much for them”. For the same reason, I did not ask my parents to take the test. I didn’t want to put them in a difficult position, also in relation to possible feelings of guilt for having transmitted me the mutation.”* (Gaia, 42 y.o., cancer diagnosis)—I
	28	*“Yes, I just think my dad has closed the chapter on that (cancer), that’s a story from the past that he’s certainly carrying it with himself, but he didn’t want it to be present anymore. It’s probably wrong (of him), it’s hard to describe, it’s just a very extreme story from the past. And for me it is just, that for me the genetic defect is more acute/present than for my father. But I think, as long as I’m healthy, it’s okay for my dad the way it is. And with my brother I find it very difficult (to talk to him) because he has a lot of trouble to find grip under his feet.”* (Antonia, 33 y.o., no cancer diagnosis)—I
	29	*“I think it makes a difference, because strangely enough I haven’t talked about it so much with my sister, because I’ve always been afraid of scaring her, about me or whatever. With my partner or with my circle of friends I could talk about it again very well. They took it in a completely different way.”* (Rose, 50 y.o., no cancer diagnosis)—FG
Respect of autonomy	30	*“Each case is, I think, different. And it has to do with your own experience. I think the only thing I would like to say is that I think each of us … must do what is right for the person who is.”* (Perla, 50 y.o., no cancer diagnosis)—FG
	31	*“And in the end, everyone has to decide for themselves whether they want to know or not and what to do about it. So, I am ready to act or not. That’s the thing, you have to think about it and make your mind up about it already before taking the test.”* (Daniela, 50 y.o., no cancer diagnosis)—FG
	32	*“He is in the field (of medicine) and he is not married (…) I don’t know if it is also related to the desire for children. If one knows that he can pass it on, one worries, if one has other plans, one does not. If one day he should have a daughter, he might change his mind. I had these stages, from something far away until it became too much, and I made decisions, it was indeed a path.”* (Sonia, 34 y.o., no cancer diagnosis)—I
	33	*“I struggle to understand and accept my cousins’ decision to ignore what was said (about the genetic risk) and to do nothing about it.”* (Gaia, 42 y.o., cancer diagnosis)—I

* I Interviews; ** FG Focus Groups.

**Table 4:** Interconnection between probands’ logic and individual and family characteristics.

Logics	Responsibility	Self-Preservation	Protection of Others	Respect of Autonomy
Individual and family characteristics	Emotional proximity	Geographical and emotional distance	Emotional proximity	Relative’s age and life stage
	Personal/family experience of genetic testing/illness	Personal experience of illness	Relative’s age and life stage	Relative’s health literacy
	Personality traits	Family dynamics	Family dynamics	Relative’s gender
	Relative’s gender	Personality traits	Relative’s personality traits	Emotional proximity/distance

**Table 5:** Supporting quotes about “The complexity of arbitration”.

Themes	Quotes	Supporting Quotes
The complexity of arbitration	34	*“I’m not going to upset him (my son). I just … it’s so that I don’t miss out on something and then … and then that’s it.”* (Federica, 40 y.o., no cancer diagnosis)—I *
	35	*“No, my daughter does not do any checks and does not want to do the test. (…) It’s her choice, sometimes we tell her but nobody can force her, she does what she feels. (…) On the one hand as a mother maybe I would like that … but I live this well … Maybe my daughter is a little less determined…”* (Bruna, 67 y.o., cancer diagnosis)—I
	36	*“I did not tell to my father because this will take on enormous proportions for him and me, it will add something to me.”* (Katarina, 33 y.o., no cancer diagnosis)—FG **
	37	*“I also realize with my brother that he really doesn’t want to talk about it, because with men it’s like this that the disease only comes to them when they’re in their 50s and 60s. (…) But for him it’s right at the moment that he doesn’t know and he doesn’t think about it.”* (Antonia, 33 y.o., no cancer diagnosis)—I
	38	*“It was only two years ago that I had more to do with my cousins and that I realized that the two of them didn’t know much and didn’t have much information. And yes, I felt a bit guilty afterwards, because I thought I should have informed them a lot more.”* (Gisela, 46 y.o., no cancer diagnosis)—FG
	39	*“So, I know that my cousin who...who started the whole thing (communication to relatives), she had a hard time with it. She had the impression that she...that she was dropping a bomb. She was not well for a while. Moreover, when she knew I was positive, she was afraid to see me. (…) She was afraid that I would be mad at her.”* (Federica, 40 y.o., no cancer diagnosis)—I
	40	*“I’m satisfied with what they told me... (The doctor) talked to me well …, she explained me well (…) I immediately sent the test results to my two sisters because of what Dr. G. told me to tell to my family and I also informed all the other family members.”* (Sabina, 52 y.o, no cancer diagnosis)—I
	41	*“When I was told the result, he told me that he had prepared a letter for the families, that I had to distribute. It explained what to do and that you had to approach. (…) I thought it was good, it was important, it gave importance, credit, I thought, to what was happening.”* (Christine, 47 y.o., no cancer diagnosis)—FG

* I Interviews; ** FG Focus Groups.

## Data Availability

Raw data are available upon request to Maria C. Katapodi (maria.katapodi@unibas.ch).
